# Supplementary material for: The Evolution and Characterization of the RNA Interference Pathways in Lophotrochozoa
Source: Genome Biol Evol. 2024 May 7;16(5):evae098. doi: 10.1093/gbe/evae098 (PMC11114477; doi:10.1093/gbe/evae098)
Supplement: evae098_Supplementary_Data [file evae098_supplementary_data.zip › Supplementary_materials.zip!/selected_miRNAs/NoGe_selected_miRNAs/mirdeep_pdf_files/NMRB01001254.1_Notospermus_geniculatus_strain_Ushimado_scaffold1254,_whole_genome_shotgun_sequence_26708.pdf]

Provisional ID : NMRB01001254.1_Notospermus_geniculatus_strain_Ushimado_scaffold1254,_whole_genome_shotgun_sequence_26708
Score total : 1221.8
Score for star read(s) : 3.9
Score for read counts : 1222.2
Score for mfe : -7.2
Score for randfold : 0
Score for cons. seed : 3
Total read count : 2409
Mature read count : 1585
Loop read count : 0
Star read count : 824

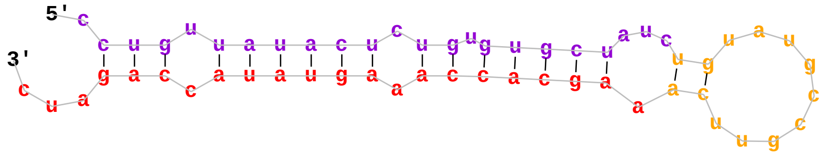

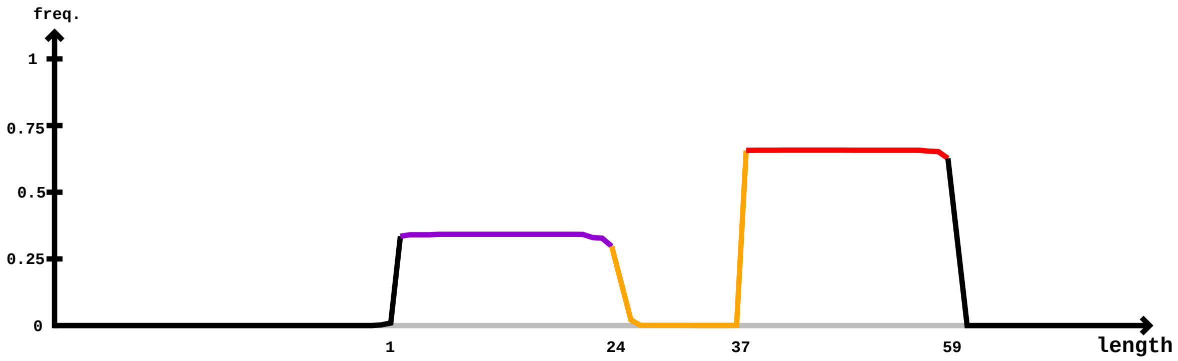

Star Mature

```
5'- aagaucuugcuuuuacuguccuugccacgugucuccuguuauacucugugugcuaucuguaugccguucaaagcaccaaaguauaccagaucacaaguucacugcacuugcu  -3'   obs
    aagaucuugcuuuuacuguccuugccacgugucuccuguuauacucugugugcuaucuguaugccguucaaagcaccaaaguauaccagaucacaaguucacugcacuugcu         exp
    .((((...((.............)).....)))).(((.((((((.((.(((((...((.........)).))))))).)))))).)))....(((((.......)))))..   reads   mm   sample
    ...............................cuccuguuauacucugugugc............................................................   1       0    Mm2
    ...............................cuccuguuauacucugugugcu...........................................................   4       0    Mm2
    ................................uccuguuauacucugugugcu...........................................................   2       0    Mm2
    ................................uccuguuauacucugugugcuau.........................................................   3       0    Mm2
    ................................uccuguuauacucugugugcuauc........................................................   1       0    Mm2
    ................................uccuguuauacucugugugcuauU.......................................................   2       1    Mm2
    ..................................ccuguuauacucugugugcu..........................................................   5       0    Mm2
    ..................................ccuguuauacucugugugcua.........................................................   3       0    Mm2
    ..................................ccuguuauacucuguguUcuau........................................................   1       1    Mm2
    ..................................ccuguuauacucugugugcuau........................................................   49      0    Mm2
    ..................................ccuguuauacucugugAgcuauc.......................................................   1       1    Mm2
    ..................................ccuguuauacucugugugcuauA.......................................................   1       1    Mm2
    ..................................ccuguuauacucugugGgcuauc.......................................................   1       1    Mm2
    ..................................ccuguuauacAcugugugcuauc.......................................................   1       1    Mm2
    ..................................ccuguuauacucuguguAcuauc.......................................................   4       1    Mm2
    ..................................ccuguuauacucugugugcuauU.......................................................   4       1    Mm2
    ..................................ccugAuauacucugugugcuauc.......................................................   1       1    Mm2
    ..................................ccuguuauacucugugugcuauc.......................................................   237     0    Mm2
    ..................................ccuguuauacucugugugcuaucA......................................................   2       1    Mm2
    ..................................ccuguuauacucugugugcuaucu......................................................   172     0    Mm2
    ..................................ccuguuauacucuAugugcuaucu......................................................   1       1    Mm2
    ..................................ccuguuauacucugugGgcuaucu......................................................   1       1    Mm2
    ..................................ccuguuauacucuguguAcuaucu......................................................   1       1    Mm2
    ..................................ccuguuauacucugugugcuaucG......................................................   2       1    Mm2
    ..................................ccuguuauacucuguguUcuaucu......................................................   1       1    Mm2
    ..................................ccuguuauacucugugCgcuaucu......................................................   2       1    Mm2
    ..................................ccuguuauacucugugugcuaucug.....................................................   23      0    Mm2
    ..................................ccuguuauacucugugGgcuaucug.....................................................   1       1    Mm2
    ..................................ccuguuauacucugugugcuaucuguaugcc...............................................   1       0    Mm2
    ...................................cuguuauacucugugugcuauc.......................................................   3       0    Mm2
    ...................................cuguuauacucugugugcuaucu......................................................   1       0    Mm2
    ...................................cuguuauacucugugugcuaucug.....................................................   2       0    Mm2
    ...........................................................uaugccguucaaagcaccaaag...............................   1       0    Mm2
```

Star Mature

```
aagaucuugcuuuuacuguccuugccacgugucuccuguuauacucugugugcuaucuguaugccguucaaagcaccaaaguauaccagaucacaaguucacugcacuugcu
.....................................................................Caagcaccaaaguauaccagauc.................... 1 1 Mm2
......................................................................aagcaccaaaguauaccaga...................... 4 0 Mm2
......................................................................aagcaccaaaguauaccagau..................... 21 0 Mm2
......................................................................aagcaccaaaguauaccagauc.................... 796 0 Mm2
......................................................................aagcaccaaaguaCaccagauc.................... 1 1 Mm2
......................................................................aagcaUcaaaguauaccagauc.................... 1 1 Mm2
......................................................................aagcaccaaaguauaccagauU.................... 4 1 Mm2
......................................................................aagUaccaaaguauaccagauc.................... 1 1 Mm2
......................................................................aagGaccaaaguauaccagauc.................... 1 1 Mm2
......................................................................aGgcaccaaaguauaccagauc.................... 1 1 Mm2
......................................................................aagcaccaaaAuauaccagauc.................... 1 1 Mm2
......................................................................aagcaccaaaCuauaccagauc.................... 1 1 Mm2
......................................................................Uagcaccaaaguauaccagauc.................... 1 1 Mm2
......................................................................aagcaAcaaaguauaccagauc.................... 1 1 Mm2
......................................................................aagcaccaaaguauaGcagauc.................... 1 1 Mm2
......................................................................aaUcaccaaaguauaccagauc.................... 1 1 Mm2
......................................................................aagcaccaaaguauaccagUuc.................... 1 1 Mm2
......................................................................aagcaccaaaguauaccGgauc.................... 2 1 Mm2
......................................................................aagcaccaaaguaGaccagauc.................... 1 1 Mm2
......................................................................aagcacUaaaguauaccagauc.................... 1 1 Mm2
......................................................................aagcaccaaaguauaccUgauc.................... 1 1 Mm2
......................................................................aagcaccaaaguauaccCgauc.................... 4 1 Mm2
......................................................................aagcaccaaaguauacAagauc.................... 2 1 Mm2
......................................................................aagcaccaaaguauaccagGuc.................... 1 1 Mm2
......................................................................aagcaccaaaUuauaccagauc.................... 2 1 Mm2
......................................................................aagcaccaaaguauaccagauca................... 1 0 Mm2
......................................................................aagcaccaaaguauaccagaucU................... 9 1 Mm2

................................cuccuguuauacucugugugcu.......................................................... 1 0 Mm1
..................................ccuguuauacucugugugcuauc....................................................... 47 0 Mm1
..................................ccuguuauacucugAgugcuauc....................................................... 1 1 Mm1
..................................ccuguuauacGcugugugcuauc....................................................... 1 1 Mm1
..................................ccuguuauacucuguguUcuauc....................................................... 1 1 Mm1
..................................ccuguuauacucugugugcuaucu...................................................... 13 0 Mm1
..................................ccuguuauacucuguguCcuaucu...................................................... 1 1 Mm1
..................................ccugAuauacucugugugcuaucu...................................................... 1 1 Mm1
......................................uuauacucugugugcuaucug..................................................... 4 0 Mm1
......................................................................aagcaccaaaguauaccagau..................... 11 0 Mm1
......................................................................aagcaccaaaguauacAagauc.................... 1 1 Mm1
......................................................................aagcaccaaaguauaccagauc.................... 38 0 Mm1

.................................uccuguuauacucugugugcG.......................................................... 1 1 Fm2
.................................uccuguuauacucugugugcu.......................................................... 6 0 Fm2
..................................ccuguuauacucuguguUcuauc....................................................... 1 1 Fm2
..................................ccuguuauacucugugugcuauc....................................................... 44 0 Fm2
..................................ccuguuauacucugugugcuaucug..................................................... 10 0 Fm2
...................................Nuguuauacucugugugcuauc....................................................... 1 1 Fm2
...................................cuguuauacucugugugcuauc....................................................... 5 0 Fm2
......................................................................aagcaccaaaguauacUagauc.................... 1 1 Fm2
......................................................................aagcaccaaaguauacAagauc.................... 2 1 Fm2
......................................................................aagcaccaaaguauaccagauc.................... 142 0 Fm2
......................................................................aagcaccaaaAuauaccagauc.................... 1 1 Fm2
......................................................................aagcaccaaaguauaccUgauc.................... 1 1 Fm2
......................................................................Uagcaccaaaguauaccagauc.................... 1 1 Fm2

..................................ccuguuauacucugugugcuau........................................................ 5 0 Fm1
..................................ccuguuauacucugugugcuauc....................................................... 8 0 Fm1
..................................ccuguuauacucugugugcuaucu...................................................... 3 0 Fm1
..................................ccuguuauacucugugugcuaucug..................................................... 6 0 Fm1
......................................................................aagcaccaaaguauaccagau..................... 8 0 Fm1
......................................................................aagcaccaaaguauaccGgauc.................... 1 1 Fm1
......................................................................aagcaccaaaguauacAagauc.................... 1 1 Fm1
......................................................................aagcaccaaaguauaccUgauc.................... 1 1 Fm1
......................................................................aagcaccaaaguauaccagauc.................... 120 0 Fm1
......................................................................aagcaccaaaguauaccagaucU................... 4 1 Fm1

..................................ccuguuauacucugugugcu.......................................................... 1 0 Mm3
..................................ccuguuauacucugugugcua......................................................... 1 0 Mm3
..................................ccuguuauacucugugugcuau........................................................ 9 0 Mm3
..................................ccuguuauacucugugugcuauc....................................................... 41 0 Mm3
```

Star Mature

aagaucuugcuuuuacuguccuugccacgugucuccuguuauacucugugugcuaucuguaugccguucaaagcaccaaaguauaccagaucacaaguucacugcacuugcu			
..................................ccuguuauacucugugugcuauU...	2	1	Mm3
..................................ccuguuauacucugugugUuauc...	1	1	Mm3
..................................ccuguuauacucuguguUcuauc...	1	1	Mm3
..................................ccuguuauacucugugGgcuauc...	1	1	Mm3
..................................ccuguuauacucugugugcuaucG..	1	1	Mm3
..................................ccuguuauacucugugugcuaucu..	33	0	Mm3
..................................ccuguuauacucugugugcuaucug...	4	0	Mm3
...................................cuguuauacucugugugcuaucu..	1	0	Mm3
....aagcaccaaaguauaccagau.....................	5	0	Mm3
....aagcaccaaaguauacAagauc....................	1	1	Mm3
....aagcaccaaaguauaccagauc....................	200	0	Mm3
....aagcaccaaaguauaccUgauc....................	1	1	Mm3
....Nagcaccaaaguauaccagauc....................	1	1	Mm3
....aagcaccaCaguauaccagauc....................	1	1	Mm3
....aagcaccaaaguauaccagaucU...................	5	1	Mm3
....aagcaccaaaguauaccagauca...................	1	0	Mm3
....agcaccaaaguauaccagauc....................	1	0	Mm3
....accaaaguauaccagauc....................	1	0	Mm3
.................................uccuguuauacucugugugcu..	2	0	Fm3
..................................ccuguuauacucugugugcu..	6	0	Fm3
..................................ccuguuauacucugugugcua...	2	0	Fm3
..................................ccuguuauacucugugugcuau..	6	0	Fm3
..................................ccuguuauacucugugugcuauc...	15	0	Fm3
..................................ccuguuauacucugugugcuauU...	1	1	Fm3
..................................ccuguuauacucugugugcuaucu..	3	0	Fm3
....aagcaccaaaguauaccag.......................	8	0	Fm3
....aagcaccaaaguauaccagau.....................	16	0	Fm3
....aagcaccaaaguauaccagauc....................	143	0	Fm3
....aagcaccaaaAuauaccagauc....................	1	1	Fm3
....aagcaccaaaguauaccagauA....................	1	1	Fm3
....aagcaccaaaguauaccagaucU...................	3	1	Fm3
....aagcaccaaaguauaccagauca...................	1	0	Fm3
